# Evaluation of the jejunal microbiota and microbial markers in laying hens fed diets with black soldier fly larval fat as a partial or total replacement for soybean oil

**DOI:** 10.1016/j.psj.2026.107745

**Published:** 2026-09-09

**Authors:** Bartosz Kierończyk, Aleksandra Drażbo, Jerzy Juśkiewicz, Krzysztof Kozłowski, Piotr Szymkowiak, Liliana Ciesielska, Daria Praska, Muhammad Rumman Aslam, Mateusz Rawski, Damian Józefiak

**Affiliations:** a Department of Animal Nutrition, Faculty of Veterinary Medicine and Animal Science, Poznań University of Life Sciences, Wołyńska 33, Poznań, 60-637, Poland; b Department of Poultry Science and Apiculture, University of Warmia and Mazury in Olsztyn, Oczapowskiego 5, Olsztyn, 10-719, Poland; c Department of Biological Functions of Food, Division of Food Science, Institute of Animal Reproduction and Food Research, Polish Academy of Sciences, Tuwima 10, Olsztyn, 10-748, Poland; d Department of Zoology, Laboratory of Inland Fisheries and Aquaculture, Faculty of Veterinary Medicine and Animal Science, Poznań University of Life Sciences, Wojska Polskiego 71c, Poznań, 60-625, Poland

**Keywords:** *Hermetia illucens*, Next-generation sequencing, Microbial activity, Microbial markers, Poultry

## Abstract

In this study, the effects of partial or complete replacement of soybean oil with *Hermetia illucens* larval (**BSF**) fat on the jejunal microbial ecosystem and microbial activity in laying hens were evaluated. Three diets were used: a soybean oil-based diet (**SO**) and two diets in which 50% (**BSF50**) or 100% (**BSF100**) of soybean oil was replaced with insect fat. Jejunal digesta were analyzed for physicochemical parameters, short-chain fatty acids (**SCFAs**), and microbial composition using 16S rRNA gene sequencing. Functional metabolic potential was predicted using PICRUSt2. Dietary treatments did not influence (*P* > 0.05) digesta weight, dry matter, viscosity, pH, total SCFA levels, or microbial alpha diversity. However, the inclusion of BSF fat significantly reduced putrefactive short-chain fatty acids, mainly through a decrease (*P* < 0.001) in isobutyric acid content, while the iso-valeric acid content increased (*P* = 0.011) under complete replacement. The relative proportion of acetic acid increased (*P* = 0.002), whereas that of propionic acid decreased (*P* < 0.001) compared with that in the group fed the soybean oil diet. The complete replacement of soybean oil promoted shifts (*P* < 0.05) toward Firmicutes-associated fermentative and spore-forming taxa, including *Bacillus, Paenibacillus*, and several Clostridia-related lineages, whereas the presence of soybean oil favored (*P* < 0.05) the growth of *Lactobacillus reuteri*. Biomarker analysis (**LEfSe**) supported these patterns, revealing the enrichment of Clostridia-related taxa under partial substitution and Bacillales- and Paenibacillales-associated taxa under complete substitution. Functional prediction using PICRUSt2 indicated that BSF fat inclusion was associated with a higher (*P* < 0.05) predicted representation of pathways related to bile acid biosynthesis and carbohydrate metabolism, and a lower (*P* < 0.05) predicted representation of pathways associated with oxidative phosphorylation and certain lipid and amino acid metabolic processes. Overall, replacement of soybean oil with *H. illucens* larval fat modulated the jejunal microbiota and its predicted metabolic potential without disrupting overall microbial diversity or fermentation intensity, indicating that insect-derived lipids may serve as alternative dietary fat sources capable of shaping the jejunal microbial ecosystem of laying hens.

## Introduction

The growing interest in insect-based feed materials for poultry nutrition has expanded opportunities for more sustainable meat and egg production. To date, research has focused primarily on meeting protein requirements through full-fat or defatted meals derived from various invertebrates, particularly *Hermetia illucens* (the black soldier fly, **BSF**), which is currently the most commercially viable dipteran species worldwide (Kierończyk et al., 2022a). The chemical composition of BSF biomass, together with its functional properties linked to antimicrobial peptides, lauric acid, and prebiotic chitin, supports its practical application in poultry diets (Kierończyk et al., 2022a). The impact of BSF biomass inclusion on gastrointestinal tract (**GIT**) microecology has been extensively investigated in broiler chickens (Beller et al., 2024), turkey poults (Sypniewski et al., 2020), laying hens (Mahayri et al., 2025) and Japanese quails (Atallah et al., 2023). In contrast, substantially fewer studies have focused specifically on insect-derived fat as an alternative dietary energy source. Sosa and Fogliano (2017) demonstrated that insect fatty acid (**FA**) profiles are intermediate between those of plant- and animal-derived fats, while Kierończyk et al. (2023) further reported that BSF larval fat more closely resembles animal fats because of its high saturated fatty acid (**SFA**) content. Lauric acid (C12:0), the predominant SFA in BSF fat, is a medium-chain fatty acid (**MCFA**) associated with growth-promoting and antimicrobial properties (Çenesiz and Çiftci, 2020). Consistent with this FA profile, BSF larval fat exhibits antimicrobial activity against poultry-associated pathogens, including *Campylobacter* spp. (Silvan et al., 2025). Additionally, solvent-extracted and crude BSF fat demonstrated antiparasitic activity against *Eimeria tenella in vitro* (Sedano et al., 2025). Despite these functional attributes, information regarding the effects of BSF larval fat on small intestinal microbial communities remains limited. Nevertheless, whereas the ceca are recognized as the primary site of microbial fermentation in chickens, the jejunum represents a biologically relevant site for investigating the effects of dietary fat sources because it is directly exposed to dietary lipids and is a major site of fat digestion and FA absorption (Tancharoenrat et al., 2014). Consequently, differences in the availability and absorption of FAs may influence the composition and metabolic activity of the jejunal microbial community (Jing et al., 2022), providing information complementary to that obtained from analyses of the cecal microbiota.

Hitherto, most research has focused on the cecal microbiota, while the upper GIT remains relatively underexplored. Khan et al. (2024) reported no major effects of BSF larval fat on cecal *α*-diversity or genus-level abundance in laying hens, although microbial variability differed between treatments, suggesting that dietary effects may be more strongly associated with insect meal than with fat inclusion. Nevertheless, beneficial shifts, including the increased abundances of butyrate-producing *Subdoligranulum* spp. and *Fusicatenibacter* spp., have been reported following BSF fat application (Atallah et al., 2023). Moreover, Kierończyk et al. (2022b) reported no significant changes in selected jejunal microbiota populations in broiler chickens, although decreases in digesta pH and acetic acid concentration and an increase in butyric acid concentration were observed when soybean oil (**SO**) was completely replaced with BSF larval fat. Conversely, Sypniewski et al. (2020) reported the suppression of Enterobacteriaceae in the jejunum of turkeys fed diets containing BSF larval fat. These findings indicate that responses may vary depending on bird species, age, and intestinal segment.

The inconsistent effects of lauric acid-rich BSF larval fat on jejunal and cecal microbiota, particularly when BSF larval fat is compared with SO (Kierończyk et al., 2022b; Schafer et al., 2023), highlight the need for further targeted investigation. Lauric acid is considered more biologically active in the hindgut because of monolaurin release, whereas the small intestine represents the first site of direct exposure to dietary BSF larval fat (Kierończyk et al., 2022c). Therefore, the objective of the present study was to evaluate the effects of partial or complete substitution of SO with BSF larval fat in laying hen diets on jejunal microecology, with a particular emphasis on microbial composition and short-chain fatty acid concentrations.

## Materials and methods

### Ethics statement

In accordance with Polish law and the European Union directive (No. 2010/63/EU), the trial conducted in this study did not require approval from the Local Ethics Committee for Animal Experiments. All procedures adhered to the guidance for animal experimentation and the care of the animals under study. No action involving pain or suffering was practiced, and all analyses were performed on samples collected postmortem. Animal sacrifice performed exclusively to obtain organs or tissues was not classified as a procedure. The study adhered to the ARRIVE guidelines, ensuring ethical conduct and compliance with relevant regulations, with all methods, experimental design, sampling, and results analysis reported accordingly.

### Experimental factors

The production conditions for insect-derived fat were established following the methodology described by Kierończyk et al. (2022c). In summary, the rearing substrate was free of contaminants or animal by-products, in accordance with European Union (**EU**) Regulation 2009/1069. The diet was formulated with wheat middling and a mixture of fresh vegetables and fruits. The primary feed materials included apples, carrots, potatoes, and cabbage which were mixed in equal proportions. The substrate dry matter (**DM**) content was maintained at 220 g/kg. All the ingredients were ground (2000 rpm for 1 min) to pass through a 2-mm screen before being provided *ad libitum* to the BSF. The larvae were harvested on the 10th day of rearing, subsequently sieved through a 3-mm screen, and washed at 90 °C for 10 minutes. Throughout the production process, the environmental conditions were strictly controlled, maintaining a temperature of 28 °C and a humidity level of 70%. Prior to fat extraction, the larvae were air-dried under controlled conditions (50 °C for 24 hours) via an SLN 240 dryer (POL-EKO Aparatura, Wodzisław Śląski, Poland). The mechanical extraction of BSF fat was performed with an AP 14/22 oil press (Reinartz, Neuss, Germany) through a cold-press extraction process. The extraction parameters were set at 45 °C with a humidity level of 95%. No refinement of the extracted fat was applied before its use in the experiment. The FA compositions of the dietary fat sources are presented in Table 1.

**Table 1:** Fatty acid profiles of the dietary fats used in the study, g/100 g FAs.

Fatty acids	Treatment	
	SO	BSF
Saturated fatty acids		
C8:0 Caprylic acid	-	0.07
C10:0 Capric acid	-	1.16
C12:0 Lauric acid	-	41.9
C14:0 Myristic acid	0.10	15.9
C16:0 Palmitic acid	11.1	14.2
C17:0 Heptadecanoic acid	-	0.15
C18:0 Stearic acid	4.01	2.22
C20:0 Arachidic acid	0.18	0.08
C21:0 Heneicosanoic acid	-	0.15
C22:0 Docosanoic acid	0.27	0.06
Monounsaturated fatty acids		
C14:1 Myristoleic acid	-	0.17
C16:1 Palmitoleic acid	0.13	2.27
C17:1 Heptadecenoic acid	-	0.13
C18:1 Oleic acid	23.6	17.1
C20:1 Eicosenoic acid	0.10	-
Polyunsaturated fatty acids		
C18:2 n6 Linoleic acid	51.3	1.37
C18:3 n3 Linolenic acid	7.11	0.29
C18:3 n6 y-linolenic acid	2.03	2.12
C18:4 n-3 Stearidonic acid	-	0.04
C20:5 n-3 Eicosapentaenoic acid	-	0.69
Summarized fatty acids		
SFA	15.7	76.0
UFA	84.3	24.2
MUFA	23.8	19.7
PUFA	60.4	4.51
PUFA/SFA	3.85	0.06

Abbreviations: FAs, fatty acids; SO, soybean oil; BSF, *Hermetia illucens* larval fat; SFAs, saturated fatty acids; UFAs, unsaturated fatty acids; MUFAs, monounsaturated fatty acids; PUFAs, polyunsaturated fatty acids.

### Animals and diets

The experiment was conducted at the experimental poultry farm of the Department of Poultry Science and Apiculture, University of Warmia and Mazury in Olsztyn, Poland. The study involved 168 Hy-Line Brown laying hens, aged 56 weeks, sourced from a local commercial flock. Prior to the experiment, all the birds were individually weighed and housed in three-tier battery cages (40 × 35 × 60 cm) with a 12° floor slope, accommodating two hens per cage. The hens were randomly distributed into one of three treatment groups, with 28 replicates per treatment and two hens per replicate. To minimize potential cage-level effects, the replicates were evenly distributed across the three tiers in the cage. The ambient temperature was maintained between 20 and 22 °C, and the lighting schedule was set to 16 h of continuous light followed by 8 h of darkness. The birds had *ad libitum* access to both feed and water throughout the study. The trial commenced when the hens were 56 weeks old and continued for 12 weeks, ending at 68 weeks of age.

Throughout the experiment, the birds were fed isonitrogenous and isocaloric diets in mash form that incorporated different energy sources. The experimental design was structured as follows: SO, 100% soybean oil (no replacement); **BSF50**, a 50:50 blend of cold-extracted *H. illucens* larval fat and soybean oil; and **BSF100**, 100% cold-extracted *H. illucens* larval fat (total replacement). All the diets were formulated to meet the nutrient requirements of laying hens (Hy-Line International, 2024). The composition of the experimental diets is presented in Table 2.

**Table 2:** Composition and nutrient content of the experimental diets, g/kg.

Items	Treatments^a^		
	SO	BSF50	BSF100
Feed ingredients			
Wheat	373.1	373.1	373.1
Maize	200.0	200.0	200.0
Soybean meal	89.0	89.0	89.0
Rapeseed meal	60.0	60.0	60.0
Sunflower meal	60.0	60.0	60.0
Alfalfa	50.0	50.0	50.0
Soybean oil	50.0	25.0	-
*Hermetia illucens* larval fat	-	25.0	50.0
Sodium chloride	3.6	3.6	3.6
Limestone	94.5	94.5	94.5
Monocalcium phosphate	11.9	11.9	11.9
L-lysine HCl	1.4	1.4	1.4
DL-Methionine	1.5	1.5	1.5
Vitamin-mineral premix^b^	5.0	5.0	5.0
Calculated nutritional value			
AME, kcal/kg	2700	2700	2700
Crude protein	160.0	160.0	160.0
Crude fiber	46.6	46.6	46.6
Lysine	7.50	7.50	7.50
Arginine	9.39	9.39	9.39
Methionine	4.12	4.12	4.12
Threonine	5.56	5.56	5.56
Tryptophan	1.95	1.95	1.95
Ca	39.0	39.0	39.0
Available P	4.00	4.00	4.00

a SO, 100% soybean oil; BSF50, a 50:50 blend of cold-extracted *H. illucens* larval fat and soybean oil; BSF100, 100% cold-extracted *H. illucens* larval fat.

b Supplied per kg of diet: 8 000 IU vit A, 2 500 IU vit D_3_, 20 mg vit E, 1.0 mg vit K_3_, 1.5 mg vit B_1_, 4 mg vit B_2_, 1.0 mg vit B_6_, 0.02 mg vit B_12_, 0.1 mg biotin, 6.0 mg pantothenic acid, 65.0 mg Mn from manganese oxide, 52 mg zinc from zinc oxide, 45.0 mg I from ethylene diamine dihydroiodide, 0.15 mg Se from sodium selenite, 6 mg Cu.

### Sample collection

At the end of the trial, nine birds from each treatment group were selected at random, one from each of nine randomly selected chosen cages (*n* = 9 per treatment), and sacrificed at the Department’s slaughterhouse. The birds were electrically stunned (400 mA; 350 Hz), suspended on a shackle line, and exsanguinated through a unilateral neck cut, with the right carotid artery and jugular vein severed. The digestive tract was excised, and digesta samples from the jejunum were collected, weighed, placed directly in Eppendorf tubes, and immediately frozen for microbial analysis. The jejunum was defined as the intestinal segment between the end of the duodenal loop and Meckel’s diverticulum.

The collected intestinal content samples were analyzed for DM content and viscosity. The DM content of the jejunal samples was determined at 105 °C. Jejunal digesta samples were vortexed and centrifuged at 7,211 × g for 10 minutes. A 0.5-mL aliquot of the supernatant was then transferred to a Brookfield LVDV-II+ cone-plate rotational viscometer (CP40; Brookfield Engineering Laboratories, Stoughton, MA, USA), and the viscosity was measured at a constant temperature of 39.7 °C and a shear rate of 60/s. The results are expressed as apparent viscosity.

### Microbial activity

Directly after euthanasia, the pH of the jejunal contents was measured via a microelectrode and a pH/ION meter (model 301; Hanna Instruments, Vila do Conde, Portugal). Short-chain fatty acids (**SCFAs**) were analyzed by gas chromatography (Shimadzu GC-2010, Kyoto, Japan) via a capillary column (SGE, BP21, 30 m × 0.53 mm, SGE Europe Ltd., Kiln Farm Milton Keynes, UK). The digesta samples (0.2 g) were mixed with 0.2 mL of formic acid, diluted with deionized water, and centrifuged at 7211 × g for 10 min. The supernatant was loaded onto a capillary column (SGEBP21, 30 m × 0.53 mm) via an on-column injector.

### DNA extraction, amplification, and sequencing and metagenomic analysis

Total DNA was extracted from 300 ± 10 mg of jejunal digesta samples collected from six individual birds randomly selected from the nine birds previously sacrificed in each treatment group (*n* = 6) via a Genomic Mini AX Bacteria + Kit (A&A Biotechnology, Gdynia, Poland) according to the manufacturer’s protocol. The digesta samples were mechanically lysed via a FastPrep-24 instrument (MP Biomedicals, Santa Ana, CA, USA) and Lysing Matrix A containing a garnet matrix and one 1/4″ ceramic sphere (MP Biomedicals, Santa Ana, CA, USA). After isolation, the DNA was further purified via an anti-inhibitor kit (A&A Biotechnology, Gdynia, Poland). The presence of bacterial DNA was detected via real-time PCR on an Mx3000P thermocycler (Stratagene, USA) with SYBR Green as the fluorochrome. To amplify 16S rDNA, the following universal reaction primers were used: 1055F (5′-ATGGCTGTCGTCAGCT-3′) and 1392R (5′-ACGGGCGGTGTGTAC-3′). The temperature of the reaction was set as follows: (i) 3 min at 95 °C, (ii) 15 s at 95 °C, (iii) 30 s at 58 °C, 30 s at 72 °C, and (iv) Tm 65 °C to 95 °C. Bacterial DNA was quantified via a microvolume UV–Vis spectrophotometer (NanoDrop^TM^ One, Thermo Fisher Scientific, Waltham, Massachusetts, USA) and standardized at 5 ng/μL.

16S rDNA sequencing analysis was conducted by GENOMED S.A. (Warsaw, Poland). Briefly, the microbiota diversity was determined by sequencing the amplified V3-V4 region of the 16S rRNA gene with the following primers: 16S Amplicon PCR Forward Primer (5′ TCGTCGGCAGCGTCAGATGTGTATAAGAGACAGCCTACGGGNGGCWGCAG) and 16S Amplicon PCR Reverse Primer (5′ GTCTCGTGGGCTCGGAGATGTGTATAAGAGACAGGACTACHVGGGTATCTAATCC). The amplification conditions were as follows: 3 min at 95 °C, 30 s at 95 °C (25 cycles), 30 s at 55 °C, 30 s at 72 °C, 5 min at 72 °C, and holding at 4 °C. The expected size of a Bioanalyzer trace after the amplicon PCR step was ≈ 550 bp. The PCR products were cleaned with AMPure XP beads. The libraries were subsequently sequenced by running 2 × 300 bp paired-end reads. The PCR products were cleaned, and the library was combined with the sequencing adapters and dual indices with the Nextera XT Index Kit (Illumina, San Diego, USA) according to the 16S Metagenomic Sequencing Library Preparation instructions (Illumina, San Diego, USA). The conditions for the PCR assay with the Nextera XT Index Primer were as follows: 3 min at 95 °C, 30 s at 95 °C (eight cycles), 30 s at 55 °C, 30 s at 72 °C, 5 min at 72 °C, and holding at 4 °C. Next, to purify the PCR products, AMPure XP beads were used. The library was valid to the expected size on a Bioanalyzer, for a final library of ≈ 630 bp. The libraries were quantified with a fluorometric quantification method using dsDNA-binding dyes. Individual concentrations of the DNA libraries were calculated in nM on the basis of the size of the DNA amplicons as determined by an Agilent 2100 Bioanalyzer (Agilent Technologies, Santa Clara, CA, USA).

For sequencing, the individual libraries were diluted to 4 nM, denatured with 10 mM Tris (pH 8.5) and spiked with 20% (v/v) PhiX. A 5-μL aliquot of diluted DNA was mixed for pooling the library preparations for MiSeq (Illumina, San Diego, USA) runs. The sample reads were >100,000.

The microbiome sequences were classified according to the V3 and V4 amplicons and analyzed via the 16S rRNA database. The specific sequences 341F and 785R were used for amplification and library preparation. PCR was performed with Q5 Hot Start High-Fidelity 2X Master Mix, and the reaction conditions followed the manufacturer’s requirements. Sequencing was performed on a MiSeq sequencer using paired-end (PE) technology, 2 × 250 nt, and an Illumina v2 kit. Automated initial data analyses were performed on the MiSeq apparatus via MiSeq Reporter (MSR) v2.6 software (Illumina, San Diego, USA). The analysis consisted of two stages: automatic demultiplexing of the samples and the generation of FASTQ files containing raw reads. The sequencing output consisted of read classification at several taxonomic levels: kingdom, phylum, class, order, family, genus, and species. Quality analysis of the sequences was conducted with quality control and filtering to obtain high-quality sequences. Valid sequences were screened from samples according to the barcodes at both ends of the sequences and corrected for direction by the primer sequences. Bioinformatic analysis for species-level taxonomic classification was conducted using the QIIME 2 platform with the SILVA 138 reference database (Yilmaz et al., 2014; Bolyen et al., 2019). Sequence denoising and error correction were performed using the DADA2 algorithm to resolve true biological sequences and generate amplicon sequence variants (ASVs). Taxonomic assignment was carried out across multiple hierarchical levels, including kingdom, phylum, class, order, family, genus, and species. Relative abundance profiles of the ileal microbiota were subsequently calculated on the basis of ASV abundances within each experimental group.

### Functional prediction (PICRUSt2)

The functional potential of the microbial communities was predicted using **PICRUSt2** (Phylogenetic Investigation of Communities by Reconstruction of Unobserved States 2; version 2.4.1) in a Linux bash environment. Amplicon sequence variants (**ASVs**) were generated in QIIME 2 (version 2025.4) using the DADA2 algorithm and exported along with the feature table for downstream analysis. Representative ASV sequences were placed into the reference phylogeny using SEPP, and gene family abundances were inferred using hidden-state prediction. The predicted functional profiles were annotated against the Kyoto Encyclopedia of Genes and Genomes (**KEGG**) database, generating KEGG Orthology (**KO**) abundances. Metagenome predictions were normalized by the predicted 16S rRNA gene copy number, filtered using a maximum NSTI threshold of 2.0, and collapsed into KEGG pathways using the PICRUSt2 pathway pipeline. The predicted KEGG pathway abundances were normalized to the relative abundances (proportion of total pathway counts per sample) prior to downstream statistical analyses. Photosynthesis-related KEGG pathways were excluded because they are biologically irrelevant in broiler hosts, and the results were summarized by reporting the top 10 biologically relevant pathways to improve interpretability.

### Phylogenetic affiliation and statistical analyses

The experimental design employed complete randomization. The individual bird was considered the experimental unit for SCFA concentrations, bacterial enzyme activity, digesta weight, dry matter, viscosity, and pH, with nine replicates per treatment (n = 9), whereas six birds selected from these nine replicates per treatment (*n* = 6) were used for NGS-based microbiota analyses. RStudio (2024.12.1 + 563; Posit, PBC, Boston, MA, USA) was used for the statistical analyses. The normality and homogeneity of variance of the data were assessed via the Shapiro‒Wilk test and Levene's test, respectively. For normally distributed data, one-way ANOVA and the Duncan post hoc test were used to compare multiple treatments, whereas nonnormally distributed data were analyzed via the Kruskal‒Wallis test followed by Dunn's test with Benjamini‒Hochberg adjustment. When the assumption of homogeneity was violated, Welch’s ANOVA was applied, followed by the Games–Howell post hoc test. For microbial abundance analyses, statistical comparisons were not performed across all taxa detected in the sequencing dataset. Instead, at each taxonomic level, analyses were restricted to the most abundant taxa. For each selected taxon, the statistical analysis tested whether its relative abundance differed among the three dietary treatments (SO, BSF50, and BSF100), rather than comparing abundance among taxa. Differences were considered significant at P < 0.05, while *P*-values between 0.05 and 0.10 were considered indicative of a tendency.

Further analyses of *α*-diversity (diversity within samples) and *β*-diversity (similarity between different microbiome profiles) were performed with the vegan package (v2.6-8). Four *α*-diversity indices were calculated, namely, the Shannon, Simpson, Chao1, and evenness indices. *β*-diversity was assessed via the Bray‒Curtis, Canberra, and Euclidean dissimilarity matrices and evaluated for the multivariate effects of experimental treatments on species-level bacterial communities via nonparametric permutational multivariate analysis of variance (PERMANOVA) with *adonis2* with 999 permutations (for significant differences in community composition across groups) and pairwise Adonis (pairwise comparisons between specific groups) functions, whereas bacterial profiles at the genus level among the different groups were visualized via principal coordinate analyses (**PCoA**). The differential abundance of the jejunal microbiota was determined via linear discriminant analysis effect size (**LEfSe**) with the microbiomeMarker (v1.10.0) package. Using presample normalization of the summed values to 1e+06, we applied the Kruskal‒Wallis and Wilcoxon tests with a significance level of *P* < 0.05, along with a linear discriminant analysis (**LDA**) threshold value of 3. The results were then visually represented on the basis of the Log10 (LDA score).

## Results

### Microbial activity

No significant differences were observed in the weight (*P* = 0.815), DM content (*P* = 0.758), viscosity (*P* = 0.168), or pH value (*P* = 0.168) of the jejunal digesta (Table 3). The effects on microbial activity were particularly evident in putrefactive short-chain fatty acids (**PSCFAs**). Both experimental diets, BSF50 and BSF100, reduced (*P* = 0.003) PSCFA concentrations, primarily as a result of a significant decrease (*P* < 0.001) in iso-butyric acid levels compared with those in the control group (SO). Conversely, the iso-valeric acid concentration was significantly greater (*P* = 0.011) in the BSF100 group than in the SO and BSF50 groups. The total SCFA concentration was not affected (*P* = 0.296) by the dietary treatments. With respect to the SCFA profile, acetic acid levels significantly increased (*P* = 0.002) following the inclusion of *H. illucens* fat in the laying hens’ diets, whereas the propionic acid concentration significantly decreased (*P* < 0.001) compared with that in the SO treatment group.

**Table 3:** Effects of partial or total replacement of soybean oil with *Hermetia illucens* fat in laying hen diets on the weight, dry matter, viscosity, pH, and short-chain fatty acid concentration of the jejunum, µmol/g digesta.

Item	Treatment			SEM	*p value*
	SO	BSF50	BSF100		
Digesta weight, g/kg BW	31.1	28.6	30.7	0.544	*0.815*
Digesta DM, %	23.5	21.4	21.3	0.634	*0.758*
Digesta viscosity, mPa⋅s	2.01	2.23	2.17	0.045	*0.168*
pH	6.93	6.95	6.76	0.049	*0.168*
Short-chain fatty acid, µmol/g digesta					
Acetic acid	2.92	3.67	4.05	0.269	*0.106*
0Propionic acid	0.833	0.730	0.673	0.038	*0.089*
Isobutyric acid	0.223^a^	0.060^a^	0.028^a^	0.023	*<0.001*
Butyric acid	0.073	0.050	0.069	0.004	*0.743*
Iso-valeric acid	0.040^a^	0.062^a^	0.082^a^	0.006	*0.011*
Valeric acid	0.008	0.000	0.000	0.0026	*0.368*
PSCFA	0.271^a^	0.122^a^	0.110^a^	0.0232	*0.003*
Sum SCFA	4.10	4.57	4.90	0.286	*0.296*
SCFA profile, % of total SCFA					
Acetic acid	70.2^a^	80.1^a^	80.9^a^	1.38	*0.002*
Propionic acid	20.8^a^	16.0^a^	14.8^a^	0.80	*<0.001*
Butyric acid	1.92	1.09	1.57	0.128	*0.275*

Abbreviations: SO, 100% soybean oil; BSF50, a 50:50 blend of cold-extracted *H. illucens* larval fat and soybean oil; BSF100, 100% cold-extracted *H. illucens* larval fat; BW, body weight; DM, dry matter; SCFA, short-chain fatty acid; PSCFA, putrefactive short-chain fatty acids, i.e., the sum of C4i, C5i, C5.

a Means within a row with different superscripts differ significantly (*P* < 0.05).

### Diversity and taxonomical abundance

**Fig. 1:**
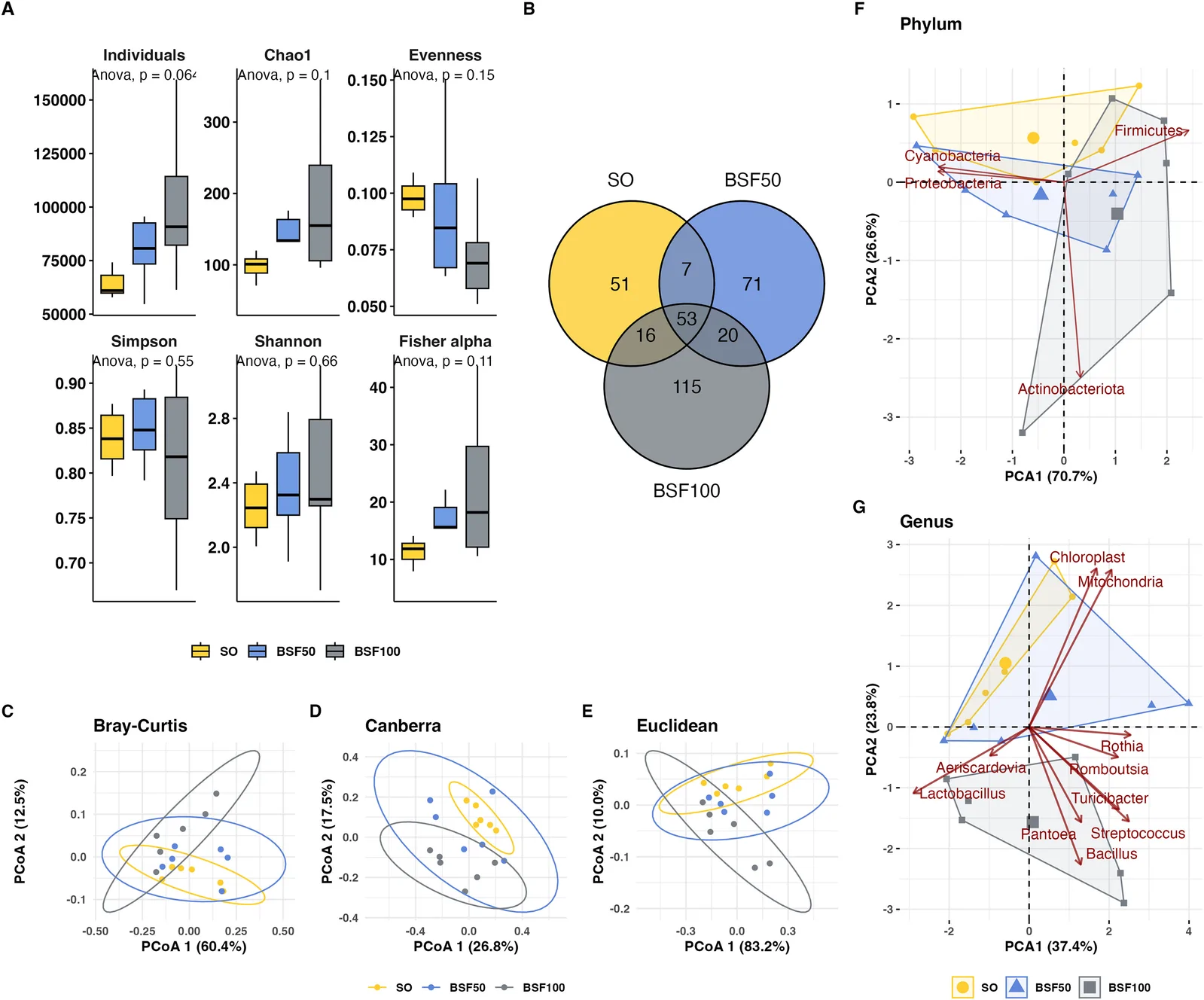
Effects of the partial or total replacement of soybean oil with *Hermetia illucens* fat in laying hen diets on selected diversity indices of the jejunal digesta, including (**A**) *α* diversity, (**B**) a Venn diagram illustrating the proportions of core operational taxonomic units (OTUs) at the genus level, (**C–E**) the results of principal coordinate analyses (PCoAs) based on different dissimilarity indices (Bray‒Curtis, Canberra, and Euclidean, respectively), and (**F‒G**) the results of principal component analyses at the phylum and genus levels, respectively. The arrows indicate correlations between the variables and dimensions. **SO**, 100% soybean oil; **BSF50**, a 50:50 blend of cold-extracted *H. illucens* larval fat and soybean oil; **BSF100**, 100% cold-extracted *H. illucens* larval fat.

**Fig. 2:**
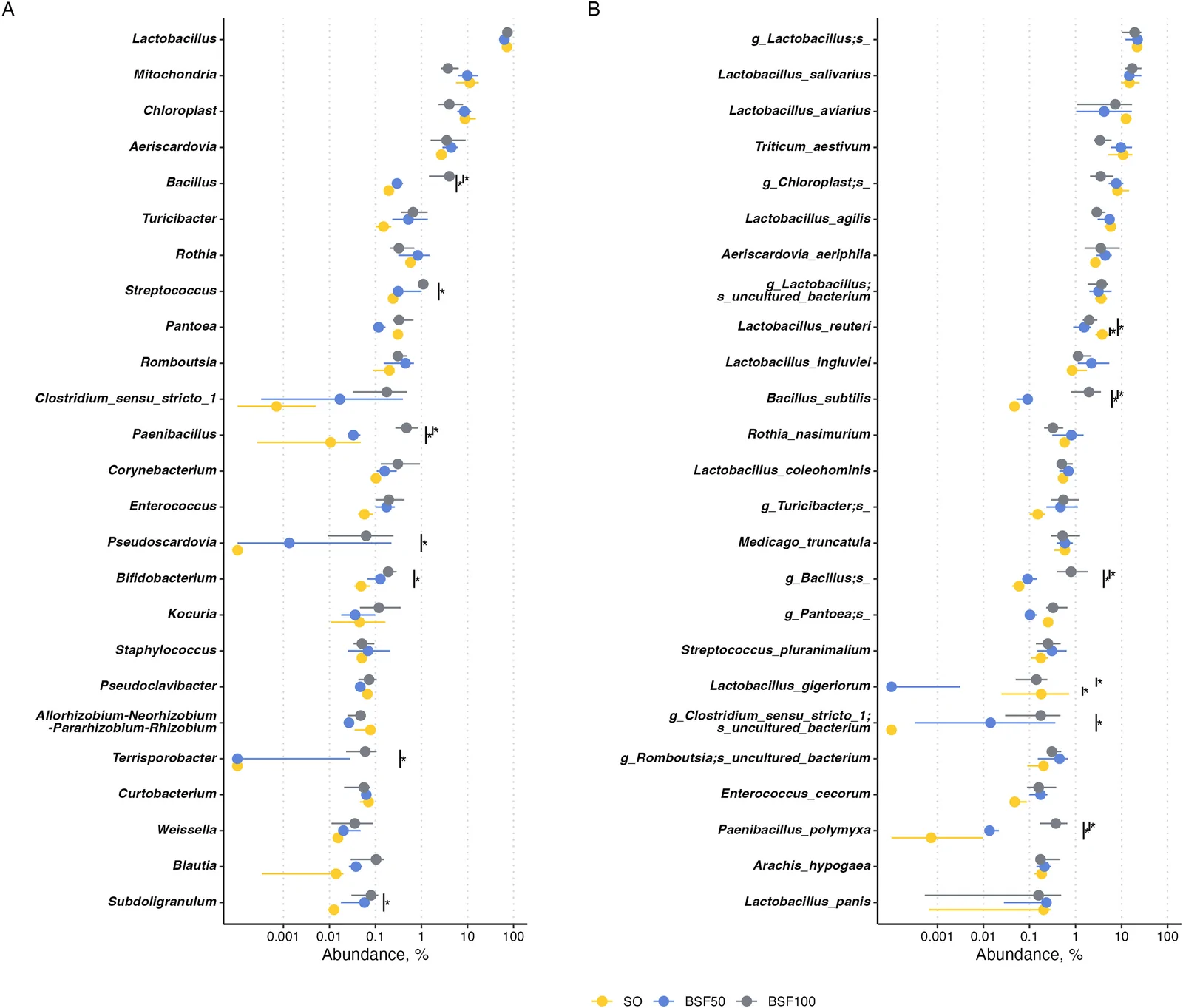
Effects of the partial or total replacement of soybean oil with *Hermetia illucens* fat in laying hen diets on the selected and most abundant jejunal digesta microbiome at the (**A**) genus and (**B**) species levels. Dots represent the medians, and lines indicate the range between the first and third quartiles (IQRs). Asterisks indicate a significant difference between treatments. **SO**, 100% soybean oil; **BSF50**, a 50:50 blend of cold-extracted *H. illucens* larval fat and soybean oil; **BSF100**, 100% cold-extracted *H. illucens* larval fat.

The selected diversity indices are presented in Fig. 1. No significant differences in *α*-diversity, including the individual (*P* = 0.064), Chao1 (*P* = 0.100), Evenness (*P* = 0.146), Shannon (*P* = 0.658), Simpson-1 (*P* = 0.549), or Fisher’s alpha (*P* = 0.111) indices, were noted (Fig. 1A). A Venn diagram illustrates that the proportion of unique taxa at the genus level reached 15.3%, 21.3%, and 34.5% for the SO, BSF50, and BSF100 treatments, respectively. The percentage of taxa shared across all the treatments was 15.9% (Fig. 1B). Significant differentiation in *β*-diversity was observed in only the PCoA computed with the Canberra dissimilarity index, with a significant difference between the SO and BSF100 groups (*P* = 0.004), in contrast to the Bray‒Curtis (*P* = 0.355) and Euclidean (*P* = 0.421) matrices (Fig. 1C-E). Microbial variability was explained in the principal component analyses (**PCA**) graphs at the phylum and genus taxonomical levels (Fig. 1F and G). The most distinct variation was observed in Firmicutes and Actinobacteriota, which were associated with BSF100 treatment, whereas Cyanobacteria and Proteobacteria were associated with SO and BSF50 treatments. At the genus level, most correlation arrows were oriented toward BSF100 treatment, with an emphasis on *Bacillus, Streptococcus, Pantoea*, and *Turicibacter*, whereas SO treatment was associated with *Lactobacillus*, and both SO and BSF50 treatments were associated with *Chloroplast* and *Mitochondria*. With respect to microbial abundance (Table 4), no significant changes were observed at the phylum level for Firmicutes (*P* = 0.351), Proteobacteria (*P* = 0.176), Cyanobacteria (*P* = 0.167), or Actinobacteriota (*P* = 0.053) after the partial or total replacement of soybean oil with *H. illucens* larval fat. However, the abundances of Clostridia (*P* = 0.015) and Coriobacteriia (*P* = 0.007) were significantly greater in the jejunal digesta in the BSF100 group than in the SO group. A similar trend was noted for the orders Bacillales (*P* = 0.003) and Clostridiales (*P* = 0.023), with BSF50 showing no significant differences between treatments. Conversely, the order Peanibacillales was present at similar levels in the SO and BSF50 groups but significantly increased in the BSF100 group (*P* = 0.003). Compared with SO treatment, BSF50 treatment increased the abundance of Peptostreptococcales-Tissierellales (*P* = 0.047). At the family level, the abundances of all the taxa, including Streptococcaceae (*P* = 0.045), Bacillaceae (*P* = 0.002), Peptostreptococcaceae (*P* = 0.050), Peanibacillaceae (*P* = 0.003), Clostiriaceae (*P* = 0.023), and Lachnospiraceae (*P* = 0.025), were increased by the inclusion of *H. illucens* larval fat as the sole dietary fat source in the laying hens’ diets compared with that in the laying hens fed a soybean oil-based diet. Furthermore, BSF50 treatment increased (*P* = 0.050) only the Peptostreptococcaceae community to levels similar to those observed with BSF100 treatment. The abundances of the 25 most frequent genera or species of the microbiome are presented in Fig. 2. Compared with the SO and BSF50 groups, the BSF100 treatment increased the abundances of *Bacillus* (*P* = 0.003) and *Peanibacillus* (*P* = 0.003) (Fig. 2A). Moreover, the abundances of the genera *Streptococcus* (*P* = 0.034), *Pseudoscardovia* (*P* = 0.035), *Bifidobacterium* (*P* = 0.014), *Terrisporobacter* (*P* = 0.041) and *Subdoligranulum* (*P* = 0.032) were greater compared with those in the control group. In terms of species abundance (Fig. 2B), in contrast to the partial or total inclusion of *H. illucens* larval fat in the bird diet, the inclusion of soybean oil stimulated the growth of *Lactobacillus reuteri* (*P* = 0.038). However, compared with SO and BSF50 treatment, BSF100 treatment significantly increased the abundances of *Bacillus subtilis, g_Bacillus* spp. (*P* = 0.002), and *Peanibacillus polymyxa* (*P* = 0.002), as well as *Clostridium sensu stricto 1;s_uncultured bacterium* (*P* = 0.036), in contrast to its abundance in the SO group. Additionally, *Lactobacillus gigeriorum* was not detected in the BSF50 treatment samples, and a significant difference between BSF50 treatment and the other treatments was noted (*P* = 0.046). Moreover, no differences were observed between the SO and BSF100 groups.

**Table 4:** Effects of the partial or total replacement of soybean oil with *Hermetia illucens* fat in laying hen diets on the selected and most abundant jejunal digesta microbiome at the phylum, class, order, and family levels.

Item	Treatment			SEM	*p value*
	SO	BSF50	BSF100		
**Phylum level**					
Firmicutes	71.2	69.9	79.6	2.88	*0.351*
Proteobacteria	13.2	12.3	6.32	1.62	*0.176*
Cyanobacteria	11.2	10.5	5.38	1.37	*0.167*
Actinobacteriota	4.36	7.22	8.59	0.95	*0.053*
**Class level**					
Bacilli	70.9	67.9	78.0	2.94	*0.371*
Alphaproteobacteria	12.8	12.0	5.13	1.62	*0.101*
Cyanobacteria	11.2	10.5	5.38	1.37	*0.167*
Actinobacteria	4.32	7.10	8.35	0.93	*0.059*
Clostridia	0.33^a^	1.05^a^^a^	1.41^a^	0.40	*0.015*
Gammaproteobacteria	0.38	0.31	1.19	0.23	*0.181*
Coriobacteriia	0.01^a^	0.12^a^^a^	0.22^a^	0.04	*0.007*
**Order level**					
Lactobacillales	70.3	66.2	70.9	3.12	*0.821*
Rickettsiales	12.4	11.9	4.87	1.62	*0.102*
Chloroplast	11.2	10.47	5.38	1.37	*0.167*
Bifidobacteriales	2.95	5.26	6.28	0.84	*0.094*
Micrococcales	1.06	1.48	1.28	0.24	*0.792*
Enterobacterales	0.34	0.27	1.09	0.22	*0.150*
Peptostreptococcales-Tissierellales	0.20^a^	0.80^a^	0.58^a^^a^	0.14	*0.047*
Bacillales	0.22^a^	0.48^a^^a^	4.59^a^	0.73	*0.003*
Corynebacteriales	0.22	0.28	0.62	0.10	*0.364*
Erysipelotrichales	0.18	0.94	1.38	0.28	*0.076*
Paenibacillales	0.03^a^	0.08^a^	0.97^a^	0.20	*0.003*
Clostridiales	0.01^a^	0.10^a^^a^	0.41^a^	0.02	*0.023*
**Family level**					
Lactobacillaceae	69.8	64.8	69.3	3.26	*0.641*
Mitochondria	12.4	11.9	4.87	1.62	*0.102*
Chloroplast	11.2	10.5	5.38	1.37	*0.167*
Bifidobacteriaceae	2.95	5.26	6.28	0.84	*0.094*
Micrococcaceae	0.74	1.26	0.81	0.20	*0.526*
Streptococcaceae	0.34^a^	0.81^a^^a^	1.21^a^	0.17	*0.045*
Bacillaceae	0.22^a^	0.47^a^	4.57^a^	0.73	*0.002*
Microbacteriaceae	0.21	0.18	0.33	0.06	*0.949*
Peptostreptococcaceae	0.19^a^	0.79^a^	0.53^a^	0.14	*0.050*
Erysipelotrichaceae	0.18	0.94	1.37	0.28	*0.076*
Corynebacteriaceae	0.14	0.26	0.58	0.09	*0.204*
Enterococcaceae	0.11	0.51	0.30	0.12	*0.447*
Staphylococcaceae	0.08	0.14	0.14	0.04	*0.990*
Paenibacillaceae	0.03^a^	0.05^a^	0.97^a^	0.20	*0.003*
Clostridiaceae	0.01^a^	0.10^a^^a^	0.41^a^	0.23	*0.023*
Lachnospiraceae	0.05^a^	0.17^a^^a^	0.24^a^	0.03	*0.025*

Abbreviations: SO, 100% soybean oil; BSF50, a 50:50 blend of cold-extracted *H. illucens* larval fat and soybean oil; BSF100, 100% cold-extracted *H. illucens* larval fat.

a Means within a row with different superscripts differ significantly (*P* < 0.05).

### Microbial markers

**Fig. 3:**
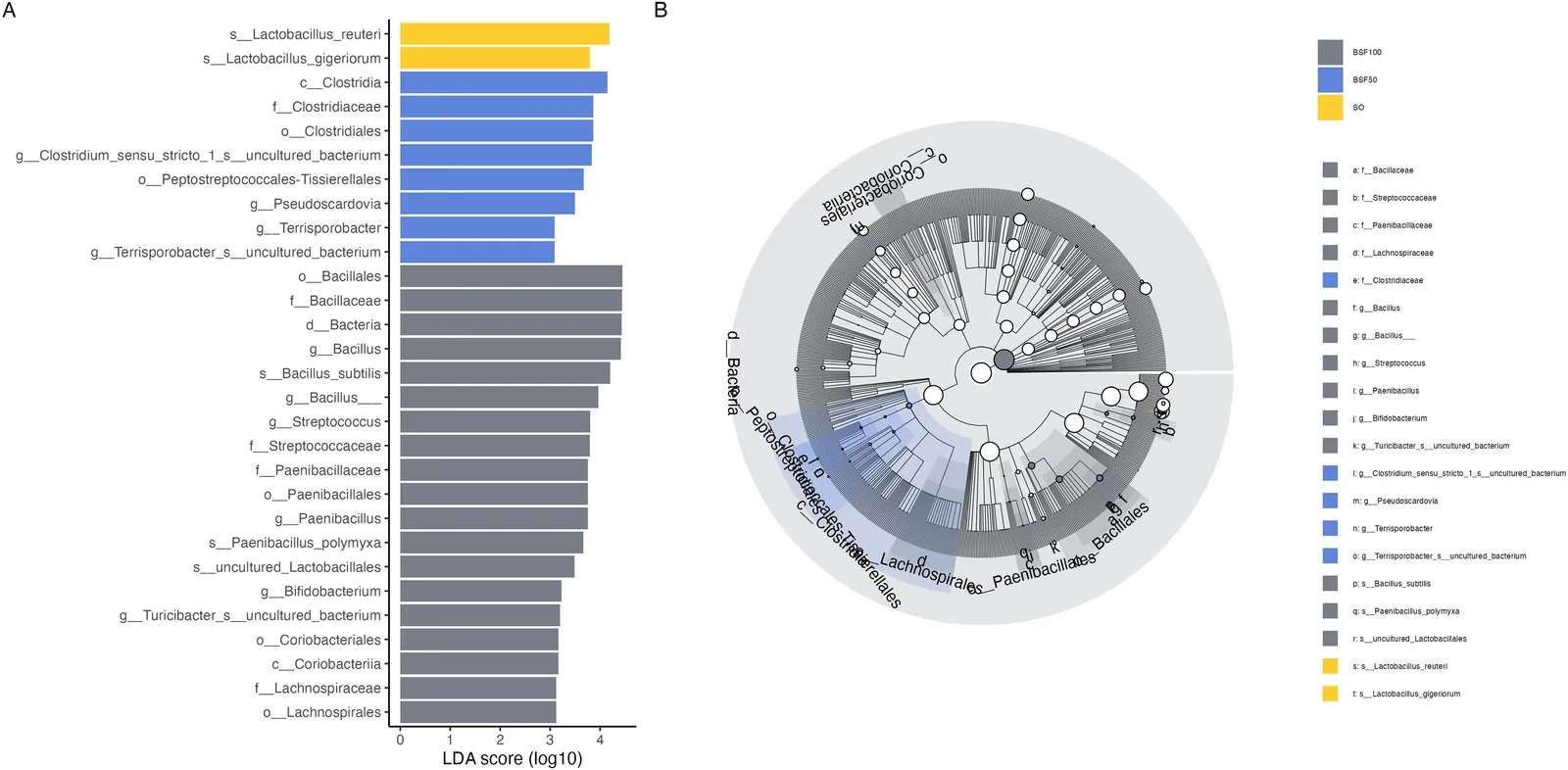
Linear discriminant analysis (LDA) scores at different taxonomic levels from three groups of broiler chickens fed different diets: domain (d), class (c), order (o), family (f), genus (g), and species (s). (**A**) Histogram plot of the LDA scores for the differentially abundant taxa among the groups. The length of the bar represents the log10-transformed LDA score. LDA scores represent bacterial taxa that are overabundant in the corresponding group. (**B**) Cladogram showing the phylogenetic relationships among different groups of organisms with significantly different levels of abundance. **SO**, 100% soybean oil; **BSF50**, a 50:50 blend of cold-extracted *H. illucens* larval fat and soybean oil; **BSF100**, 100% cold-extracted *H. illucens* larval fat.

The effect size of each marker and the phylogenetic relationships between taxa whose abundance significantly differed across the three groups are presented in Fig. 3. Only two microbial markers were identified in the SO group, namely, *Lactobacillus reuteri* (*P* = 0.038) and *Lactobacillus gigeriorum* (*P* = 0.046). In contrast, the partial replacement of SO with insect fat was associated with class Clostridia (*P* = 0.015); orders Clostridiales (*P* = 0.023) and Peptostreptococcales-Tissierellales (*P* = 0.047); family Clostridiaceae (*P* = 0.023); and the species *Clostridium sensu stricto 1;s_uncultured bacterium* (*P* = 0.036) in the microbiome*.* Furthermore, BSF50 treatment was linked to microbial markers at various taxonomic levels, including the genera *Pesudoscardovia* (*P* = 0.035) and *Terrisporobacter* (*P* = 0.041) and the species *Terrisporobacter;s_unculuters bacterium* (*P* = 0.041). The greatest number of microbial markers (19) was detected in the BSF100 group (Fig. 3A), and the number of microbial markers was related to the overall bacterial community composition, i.e., the bacterial domain (*P* < 0.001; Fig. 3B), as well as specific taxa belonging to class Bacilli. These taxa included the orders Bacillales (*P* = 0.003) and Paenibacillales (*P* = 0.003); the families Bacillaceae (*P* = 0.002), Streptococcaceae (*P* = 0.045), and Paenibacillaceae (*P* = 0.003); the genera *Bacillus* (*P* = 0.003), *Streptococcus* (*P* = 0.034), and *Paenibacillus* (*P* = 0.003); and the species *Bacillus subtilis* (*P* = 0.002)*, Paenibacillus polymyxa* (*P* = 0.002)*,* and *Turicibacter;s_uncultured bacterium* (*P* = 0.032). Additionally, the inclusion of insect fat as an energy source led to the differentiation of several abundant taxa within class Coriobacteriia (*P* = 0.007), including Coriobacteriales (*P* = 0.007), and within the class Clostridia, represented by Lachnospirales (*P* = 0.045) and the family Lachnospiraceae (*P* = 0.045). Moreover, *Bifidobacterium* (*P* = 0.014) and uncultured Lactobacillales (*P* = 0.017) were detected at the species level in the BSF100 group.

### Functional prediction

**Fig. 4:**
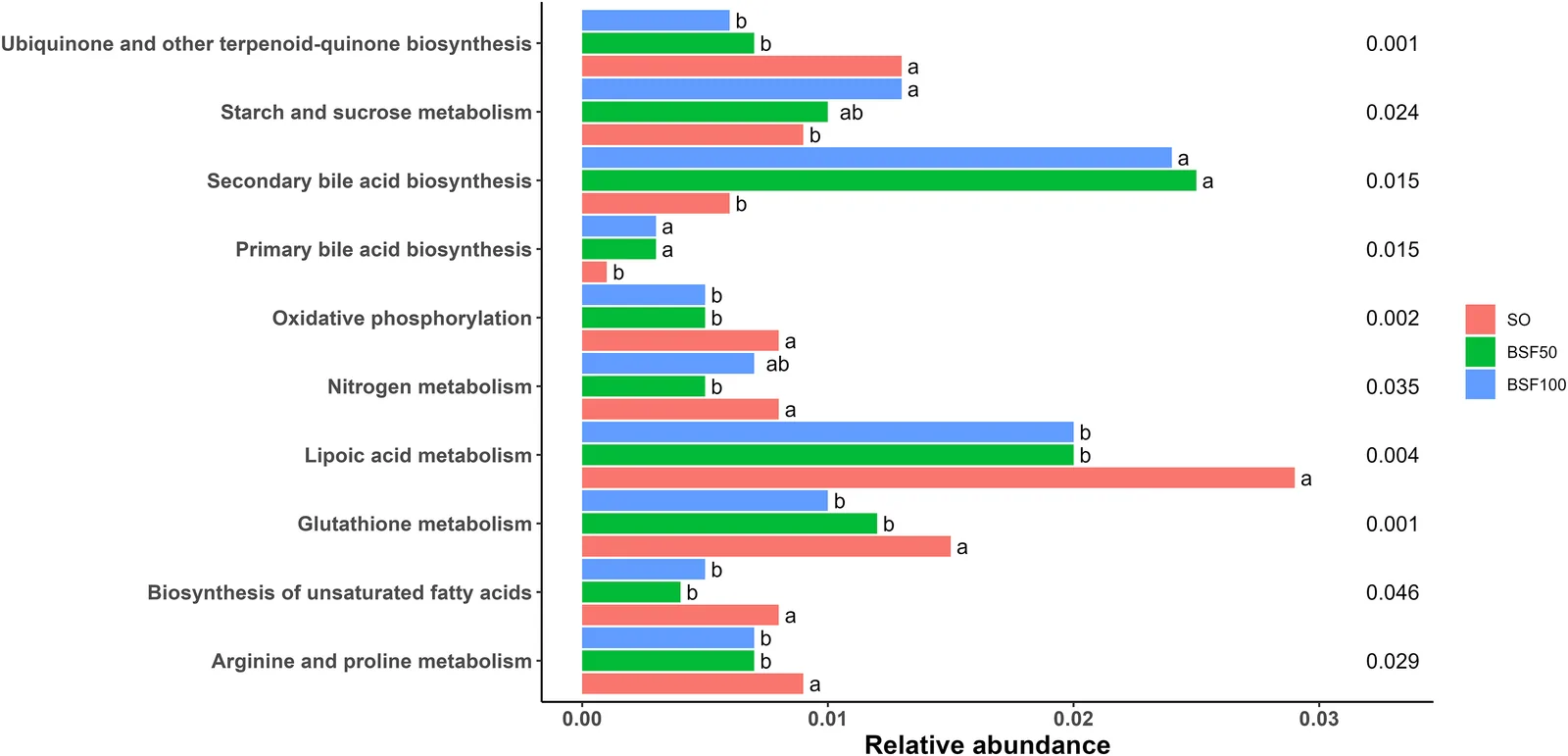
Relative enrichment of significant KEGG functional pathways predicted by PICRUSt2 in broiler chickens fed three experimental diets. **SO**, 100% soybean oil; **BSF50**, a 50:50 blend of cold-extracted *H. illucens* larval fat and soybean oil; **BSF100**, 100% cold-extracted *H. illucens* larval fat.

Functional prediction revealed significant differences in KEGG pathways among the dietary treatments. The top 10 biologically relevant pathways are presented in Fig. 4. Partial or complete replacement of SO with BSF larval fat significantly increased the predicted representation of pathways associated with primary bile acid biosynthesis (*P* = 0.015), secondary bile acid biosynthesis (*P* = 0.015), and starch and sucrose metabolism (*P* = 0.024). In contrast, the inclusion of BSF larval fat was associated with a lower predicted representation of pathways related to oxidative phosphorylation (*P* = 0.002), glutathione metabolism (*P* = 0.001), ubiquinone and other terpenoid–quinone biosynthesis (*P* = 0.001), arginine and proline metabolism (*P* = 0.029), unsaturated fatty acid biosynthesis (*P* = 0.046), and lipoic acid metabolism (*P* = 0.004). For nitrogen metabolism (*P* = 0.035), PICRUSt2 predicted a lower representation only under partial SO substitution, with no significant difference observed between the SO and BSF100 groups.

## Discussion

The effects of partial and complete SO replacement with BSF larval fat on jejunal microecology, microbial composition, and SCFA acid concentrations in laying hens were evaluated. While BSF fat was higher in SFAs, its high proportion of MCFAs may increase digestibility, because unlike long-chain SFAs, MCFAs can be directly absorbed by enterocytes. Moreover, our results indicate that the lower PSCFA concentration in the jejunal digesta of the BSF50 and BSF100 groups relative to that in the SO group was associated mainly with reduced isobutyric acid levels. This difference occurred despite an increase in isovaleric acid in the BSF100 group. PSCFAs represent a combined pool of isobutyric (C4i), isovaleric (C5i) and valeric (C5) acids. These findings differ from those of Kierończyk et al. (2022b), who reported no significant changes in PSCFAs in the jejunum or ceca of broilers fed BSF larval fat-based diets. However, partial agreement exists with their results from the cecum, which revealed higher isovaleric acid levels when SO was fully replaced with BSF fat. Moreover, the inclusion of BSF fat increased the proportional contribution (% of total SCFAs) of acetic acid while reducing that of propionic acid, although the absolute concentrations of these acids remained unchanged. A similar lack of change in absolute SCFA concentrations was reported by Kim et al. (2020), who reported no differences in ileal SCFAs when BSF fat replaced plant oils. Collectively, these observations suggest that BSF fat alters the composition of jejunal fermentation products without modifying total SCFA production. Because PSCFAs are generally derived from microbial protein fermentation, which may be associated with unfavorable gut conditions, the reduction observed here may reflect a shift in microbial metabolic activity rather than an overall change in fermentation intensity.

The dietary inclusion of BSF fat at 100% shifted the microbial composition toward Firmicutes-dominated, fermentative and spore-forming taxa**.** This shift is consistent with the antimicrobial properties of MCFAs, particularly lauric acid, which can exert selective pressure on the gut microbiota (Wu et al., 2021)**.** The increased abundance of the classes Clostridia and Coriobacteriia suggests enhanced anaerobic fermentative potential within the gut ecosystem. Members of Clostridia, particularly families such as Lachnospiraceae and Clostridiaceae, are recognized producers of butyrate and other SCFAs, which have been associated with improved villus height-to-crypt depth ratios and nutrient absorption in poultry (Onrust et al., 2015). These metabolites support epithelial energy metabolism, strengthen tight junction integrity (e.g., ZO-1 and Claudin-1), and modulate mucosal immune responses by reducing the expression of proinflammatory cytokines such as IL-8 while promoting sIgA production and regulatory T-cell activity (Liu et al., 2023a, 2023b). Such mechanisms may limit colonization by pathogens, including *Salmonella* and *Clostridium perfringens*, thereby supporting gut health and performance, which is consistent with findings from probiotic intervention studies (Liu et al., 2023a, 2023b). An increase in the abundance of Clostridia, particularly *Clostridium*_UCG-014, following the inclusion of *H. illucens* larval fat in the laying hen diets was also reported by Wu et al. (2026), who observed similar changes when 20% of soybean oil was replaced with insect-derived fat, which is consistent with the findings of the present study. In contrast, Khan et al. (2024) reported a decrease in the abundance of Clostridia when *H. illucens* larval fat was used as a dietary fat source in combination with 18% *H. illucens* larval meal as a replacement for soybean meal. However, no significant effect on Clostridia abundance was observed when *H. illucens* larval fat was used as the sole dietary fat source without the inclusion of *H. illucens* larval meal (Khan et al., 2024). It should be noted that both of these studies assessed the microbial composition in the cecal digesta, whereas the present study focused on the jejunal microbiota.

The enrichment of Peptostreptococcales-Tissierellales and Peptostreptococcaceae when BSF fat was included at either 50% or 100% suggests the possible increased protein fermentation and nitrogen turnover, which may contribute to amino acid metabolism and immune modulation. Nevertheless, the excessive proliferation of some Peptostreptococcaceae members may lead to the increased production of ammonia and other potentially detrimental metabolites, emphasizing the importance of balanced microbial shifts (Shahid et al., 2026). These findings concerning Peptostreptococcaceae are in line with those of Dabbou et al. (2021), whereas Aslam et al. (2025) reported no significant microbial changes when SO was completely replaced by BSF fat in broilers. The marked increase in Bacillus and Paenibacillus under BSF100 treatment suggests the selective proliferation of spore-forming taxa. In general, these genera are known to produce antimicrobial peptides, digestive enzymes, and immunomodulatory metabolites that improve nutrient digestibility and pathogen exclusion in poultry (Ogbuewu et al., 2022)**.** The species-level enrichment of *Bacillus subtilis* and *Paenibacillus polymyxa* further supports this interpretation, as both species are known to produce antimicrobial peptides, including bacteriocins, as well as extracellular enzymes that may contribute to the suppression enteropathogens and improved feed utilization (Wu et al., 2019). However, changes in the abundance of the genera *Bacillus* or *Peanibacillus* were not reported by Wu et al. (2026) or Khan et al. (2024), who used *H. illucens* larval fat as an energy source, nor Huang et al. (2024) or Yan et al. (2023), who evaluated the effects of live insects or insect-derived meals in laying hens. Nevertheless, these studies analyzed the cecal microbiota, where *Bacillus* and *Paenibacillus* are not among the predominant bacterial genera, whereas the present study focuses on the jejunal microbiota. The concurrent increase in Lachnospiraceae and *Terrisporobacter,* both of which are Clostridia-associated fermenters, under BSF100 treatment may indicate increased production of SCFAs, particularly butyrate, which may support intestinal development and anti-inflammatory signaling in poultry (Liao et al., 2020). The increase in the abundances of *Bifidobacterium* and *Pseudoscardovia*, which are saccharolytic Actinobacteria, suggests increased carbohydrate fermentation and lactate production, potentially facilitating cross-feeding interactions with butyrate-producing bacteria (Biavati and Santini, 2007). Moreover, probiotic species such as *Bifidobacterium bifidum* and *Bifidobacterium animalis* have been reported to increase broiler growth, antioxidant status, and intestinal barrier integrity through tight junction regulation and gut-associated lymphoid tissue (GALT) stimulation (Dev et al., 2020). However, Huang et al. (2024) reported that increasing the inclusion of live *H. illucens* larvae in laying hen diets reduced the abundance of *Bifidobacterium* in the cecal contents. A similar trend was noted by Lau et al. (2024), who reported an increased abundance of *Bifidobacterium* only in a control group of broiler chickens that did not receive diets containing *H. illucens* larval meal. Similarly, Kawasaki et al. (2019) suggested that the reduction in the cecal *Bifidobacterium* population could be attributed to *H. illucens* larval fat. In contrast, Biasato et al. (2026) reported a clear positive association between *H. illucens* larval meal and *Bifidobacterium* abundance. These divergent findings may be related, at least in part, to differences in the *H. illucens*-derived feed materials used, particularly larval meal versus larval fat, as well as the intestinal segment examined. Therefore, further investigations of the gut microecology of laying hens, particularly in the small intestine, are warranted, given the important role of the GIT segment in fat digestion and absorption. Moreover, comparisons of the microbiota between different intestinal segments, such as the cecum and jejunum, should be interpreted with caution. This is evident in the study of Kar et al. (2021), in which the abundance of *Bifidobacterium* significantly increased in the ileal digesta of pigs fed diets containing *H. illucens* larvae.

However, members of Streptococcus and the family Streptococcaceae, although common commensals in the poultry gut, may also behave as opportunistic pathogens depending on species composition. While some species, such as *Streptococcus thermophilus,* function as beneficial lactate-producing bacteria, an increase in their abundance may also reflect shifts in substrate availability or microbial niche structure (Song et al., 2022). Interestingly, *Lactobacillus reuteri* was enriched in the SO group, suggesting that soybean oil may preferentially support Lactobacilli, possibly because of differences in lipid digestion dynamics or bile-mediated microbial selection. Because *L. reuteri* may contributes to mucosal barrier integrity and produces the antimicrobial compound reuterin (Chai et al., 2023), its lower abundance in the BSF groups may indicate that lauric acid-rich lipids selectively suppress certain lactic acid bacteria while promoting spore-forming and butyrogenic taxa. In contrast, Dabbou et al. (2021) reported an increased abundance of *Lactobacillus* in broiler chickens fed *H. illucens* larval fat compared with those fed a soybean oil-containing diet. Taken together, the results obtained in the present study suggests these microbial shifts suggest that BSF fat may modulate the jejunal ecosystem through selective antimicrobial effects and by favoring metabolically resilient fermentative bacteria. However, because the microbial composition varies substantially throughout the gastrointestinal tract, the present findings reflect only the jejunal ecosystem. A comprehensive assessment of the entire gut microbiota, including the crop, gizzard and cecal sections, is necessary to determine whether the microbial shifts induced by BSF fat represent localized effects or broader alterations of the gut microecosystem.

The LEfSe (LDA > 3) results supported the differential abundance findings, indicating that the taxa enriched in each dietary group also function as discriminative microbial biomarkers. In the SO group, the enrichment of *Lactobacillus gigeriorum* and *Lactobacillus reuteri* confirms the predominance of Lactobacilli, suggesting that the microbiota is associated with lactic acid production and mucosal protection (Chai et al., 2023). In the BSF50 group, the identification of *Clostridium sensu stricto 1* (uncultured), Peptostreptococcales-Tissierellales, *Pseudoscardovia*, and uncultured *Terrisporobacter* supports a shift toward anaerobic fermentative metabolism and protein/amino acid fermentation pathways, indicating that even the inclusion of moderate amounts of BSF fat selectively promoted specific Clostridia-associated lineages (Liao et al., 2020; Shahid et al., 2026). In BSF100, the markers identified by LEfSe strongly overlapped with the abundance patterns, particularly the abundances of Bacillales/Bacillaceae (*Bacillus* and *Bacillus subtilis)* and Paenibacillales/Paenibacillaceae (*Paenibacillus* and *Paenibacillus polymyxa)*. This finding reinforces the interpretation that high BSF fat inclusion may promotes spore-forming taxa capable of producing antimicrobial compounds and extracellular enzymes (Wu et al., 2019; Ogbuewu et al., 2022). Additional markers, including Streptococcaceae (*Streptococcus*), Bifidobacterium, Turicibacter (uncultured), Coriobacteriales/Coriobacteriia, and Lachnospirales/Lachnospiraceae, suggest broader restructuring of the microbial network involving lactate producers, saccharolytic Actinobacteria, and butyrate-associated Clostridia (Biavati and Santini, 2007; Onrust et al., 2015; Liao et al., 2020; Song et al., 2022).

Functional prediction using PICRUSt2 suggested that replacing SO with BSF fat may alter the inferred metabolic potential of the jejunal microbiota, particularly in terms of pathways related to bile acid transformation and carbohydrate metabolism. The putative enrichment of predicted pathways related to primary and secondary bile acid biosynthesis may indicate differences in the inferred microbial involvement in lipid metabolism and host–microbe signaling, whereas the enrichment of pathways related to starch and sucrose metabolism may suggests differences in the inferred potential for saccharolytic activity. Conversely, the lower predicted representation of pathways related to oxidative phosphorylation and glutathione metabolism pathways may indicate differences in the inferred microbial functional potential associated with energy utilization and redox balance. A lower predicted representation of pathways related to unsaturated fatty acid, lipoic acid, and amino acid metabolism may further indicate shifts in the inferred microbial function potential associated with lipid and nitrogen metabolism under BSF fat inclusion. However, these predictions are based on inferred gene contents rather than direct functional measurements. Therefore, confirmation through whole-metagenome shotgun sequencing or complementary metatranscriptomic and metabolomic analyses is necessary to directly assess microbial gene expression and metabolic activity.

## Conclusion

Partial or complete replacement of SO with BSF larval fat modulated the jejunal microbial ecosystem of laying hens without affecting overall microbial diversity or total SCFA production. Although the inclusion of BSF fat reduced PSCFA concentrations and altered the SCFA profile, these changes likely reflected shifts in microbial metabolic processes rather than changes in overall fermentation intensity. The complete replacement of SO promoted the enrichment of Firmicutes-associated fermentative and spore-forming taxa, including *Bacillus, Paenibacillus*, and Clostridia-related lineages, which are taxa commonly associated with increased microbial resilience and metabolic versatility in the poultry gut. PICRUSt2-based functional prediction indicated differential representation of KEGG pathways related to bile acid metabolism and carbohydrate utilization. Collectively, these findings indicate that BSF fat can reshape the jejunal microbiota while maintaining overall microbial stability, supporting its potential use as an alternative dietary lipid source in laying hen nutrition.

## Funding

This work was supported by an OPUS-20 grant titled “The role of *Hermetia illucens* larvae fat in poultry nutrition—from the nutritive value to the health status of broiler chickens” (No. 2020/39/B/NZ9/00237), which was financed by the National Science Center (Poland), and statutory funding (No. 506.533.04.00) from the Faculty of Veterinary Medicine and Animal Sciences, Department of Animal Nutrition, Poznań University of Life Sciences. The publication was financed by the Polish Minister of Science and Higher Education as part of the Strategy of the Poznan University of Life Sciences for 2024–2026 in the field of improving scientific research and development work in priority research areas.

## Author contributions

**Bartosz Kierończyk** contributed to the conception and design of the study, data curation, formal analysis, methodology, resources, software, visualization, and drafting and critical revision of the manuscript. He approved the final version and agreed to be accountable for all aspects of the work.

**Aleksandra Drażbo** contributed to the conception and design of the study, data curation, formal analysis, investigation, resources, and drafting and critical revision of the manuscript. She approved the final version and agreed to be accountable for all aspects of the work.

**Jerzy Juśkiewicz** contributed to formal analysis and resources and critically revised the manuscript. He approved the final version and agreed to be accountable for all aspects of the work.

**Krzysztof Kozłowski** contributed to supervision and critically revised the manuscript. He approved the final version and agreed to be accountable for all aspects of the work.

**Piotr Szymkowiak** contributed to formal analysis and critically revised the manuscript. He approved the final version and agreed to be accountable for all aspects of the work.

**Liliana Ciesielska** contributed to formal analysis and critically revised the manuscript. She approved the final version and agreed to be accountable for all aspects of the work.

**Daria Praska** contributed to formal analysis and critically revised the manuscript. She approved the final version and agreed to be accountable for all aspects of the work.

**Muhammad Rumman Aslam** contributed to formal analysis, software, and visualization and critically revised the manuscript. He approved the final version and agreed to be accountable for all aspects of the work.

**Mateusz Rawski** contributed to formal analysis and critically revised the manuscript. He approved the final version and agreed to be accountable for all aspects of the work.

**Damian Józefiak** contributed to the conception and design of the study, funding acquisition, project administration, and supervision and critically revised the manuscript. He approved the final version and agreed to be accountable for all aspects of the work.

## Ethics declaration

This study was conducted in accordance with the ARRIVE (Animal Research: Reporting of In Vivo Experiments) guidelines. Ethics committee approval was not required. In accordance with Polish law and the European Union directive (No. 2010/63/EU), the trial conducted in this study did not require approval from the Local Ethics Committee for Animal Experiments. All procedures adhered to the guidance for animal experimentation and the care of the animals under study. No action involving pain or suffering was practiced, and all analyses were performed on samples collected postmortem. Animal sacrifice performed exclusively to obtain organs or tissues was not classified as a procedure. The study adhered to the ARRIVE guidelines, ensuring ethical conduct and compliance with relevant regulations, with all methods, experimental design, sampling, and results analysis reported accordingly.

## Disclosures

The authors declare that they have no known competing financial interests or personal relationships that could have appeared to influence the work reported in this paper.

## Data Availability

All the raw data obtained in this study are deposited in an official repository (https://doi.org/10.18150/SKLM9R).
